# Association Between Wine Consumption and Cognitive Decline in Older People: A Systematic Review and Meta-Analysis of Longitudinal Studies

**DOI:** 10.3389/fnut.2022.863059

**Published:** 2022-05-12

**Authors:** Maribel Lucerón-Lucas-Torres, Iván Cavero-Redondo, Vicente Martínez-Vizcaíno, Alicia Saz-Lara, Carlos Pascual-Morena, Celia Álvarez-Bueno

**Affiliations:** ^1^Universidad de Castilla-La Mancha, Health and Social Research Center, Cuenca, Spain; ^2^Universidad Autónoma de Chile, Facultad de Ciencias de la Salud, Talca, Chile; ^3^Universidad Politécnica y Artística del Paraguay, Asunción, Paraguay

**Keywords:** cognitive decline, wine, older people, alcohol consumption, elderly

## Abstract

**Background:**

Low-to-moderate alcohol consumption appears to have potential health benefits. Existing evidence concludes that wine may be associated with a lower incidence of certain diseases. This systematic review and meta-analysis aim to examine evidence on the association between wine consumption and cognitive decline and to analyze whether this association varies depending on the wine consumption level or is affected by individual and study characteristics, including mean age, percentage of women participants, and follow-up time.

**Methods:**

In this systematic review and meta-analysis, we undertook a search in MEDLINE (*via* PubMed), Scopus, Cochrane, and Web of Science databases for longitudinal studies measuring the association between wine consumption and cognitive decline from their inception to May 2021. Effect sizes were calculated using the DerSimonian and Laird and Hartung-Knapp-Sidik-Jonkman methods.

**Results:**

The search retrieved 6,055 articles, 16 of which were included in this systematic review. In total, 12 studies were included in the meta-analysis. The studies were published between 1997 and 2019. They were conducted in nine different countries. The sample size of the included studies ranged from 360 to 10,308 with a mean age of 70 years old. Using the DerSimoniand and Laird method, the pooled RR for the effect of wine consumption on cognitive decline was 0.72 (95% CI 0.63–0.80; *I*^2^ = 82.4%; τ^2^: 0.0154). Using the Hartung-Knapp-Sidik-Jonkman method, the RR was 0.65 (95% CI 0.52–0.79; *I*^2^ = 94,531%; τ^2^: 0.057).

**Conclusions:**

This study may show a protective effect of wine consumption against cognitive decline. However, it would be important for future research to differentiate the types of wine within consumption.

## Introduction

The consumption of alcohol and tobacco are considered unhealthy habits, harmful to health, and are related to the development of pathologies such as cardiovascular diseases (CVD), digestive system diseases, hypertension, diabetes mellitus, or cognitive deterioration among others, representing a serious problem for public health (1–3). Indeed, alcohol consumption increases the risk of dementia, especially early-onset dementia (4–10), indicating a negative impact of alcohol consumption on different areas of familial, social, and cultural wellbeing (11–13).

The alcohol consumption recommendations established by the WHO are 30 g for men and 20 g for women, 3 standard drinking units (SBUs), and 2 SBUs, respectively, as 1 SBU corresponds to 10 g of pure alcohol (14). It has been suggested that low-to-moderate alcohol consumption could be beneficial to the health of middle-aged (15) and older subjects (16–18), leading to a J-shaped or inverse U-shaped association between alcohol and cognitive function, heart disease (19, 20) and all-cause mortality (21). Furthermore, and according to previous systematic reviews, moderate alcohol consumption appears to be associated with a lower risk of cognitive impairment, dementia, Alzheimer's disease, and better cognition (9, 22), which may be linked to its effects on cardiovascular disease (22). These effects of alcohol consumption are not observed for vascular dementia (9), neither for heavy, chronic, and irregular alcohol consumption which are associated with an increased risk of cognitive impairment or dementia (13).

Further analysis of the data indicates that the effect of alcohol depends on the type of alcoholic beverage analyzed (20), distinguishing between beer, white wine, red wine, fortified wine, and spirits (23). Although wine consumption has been associated with a reduced risk of cerebrovascular disease and Alzheimer's disease (24), there is controversy as to whether these benefits are also reported for beer and other spirits (4, 25). The specific characteristics of wine could be the reason for its benefits. Wine is produced from the fermentation of grapes, and yeast is added, causing the sugars present in the grapes to be converted into ethanol, endowing wine with different nutritional properties. It has been reported that some components of wine, such as resveratrol, phenolic acids, and flavonoids, may exert positive health effects (26). Previous research has shown that these components reduce free cholesterol (27), have a cardioprotective effect (28), induce endothelial relaxation (29), activate NO synthase (30), inhibit platelet aggregation, and (31) prevent oxidation of low-density lipoproteins (LDL) cholesterol (32).

Regardless of previous research, the evidence on the association between wine consumption and the risk of cognitive decline remains inconclusive (9, 14, 22, 33). This systematic review and meta-analysis aim to examine the strength of the association between wine consumption and cognitive decline and to analyze whether this association varies depending on the wine consumption level or is affected by individual and study characteristics, including the mean age, percentage of women participants, and follow-up time.

## Methods

### Search Strategy and Study Selection

This systematic review and meta-analysis were performed according to the Cochrane Collaboration Handbook (34) and reported following the MOOSE guidelines (Meta-analysis of Observational Studies in Epidemiology) (35) this systematic review and meta-analysis were registered on PROSPERO (registration number CRD42021232060).

A systematic search was conducted in the MEDLINE (*via* PubMed), Scopus, Cochrane, and Web of Science databases from their inception to 25 May 2021. The literature search was updated on 15 February 2022. The search strategy included the following relevant terms: (1) “dementia,” “mental deterioration,” “Alzheimer's disease,” “vascular dementia,” “predementia syndromes,” and “mild cognitive impairment”; (2) “alcohol,” “wine,” “alcohol consumption,” and “wine consumption” and (3) “older,” “elderly,” “elderly people,” and “older people.” Finally, the reference list of the studies included in this systematic review was examined to identify relevant studies. Supplementary Table 1 presents the complete search strategy for MEDLINE.

### Eligibility

Eligible articles included longitudinal studies measuring the association between wine consumption and cognitive decline. The inclusion criteria were as follows: (i) subjects: general population without dementia at baseline aged 65 or over at the end of the study; (ii) outcomes: cognitive decline assessments using standardized and validated tests; (iii) study design: longitudinal studies; and (iv) studies reporting wine consumption separated from other alcoholic beverages. Studies were excluded if they: (i) were review articles, editorials, or patient case reports; or (ii) included patients with cognitive decline at baseline determined by a battery of psychometric tests or an examination by a neurologist. No language restrictions were applied to the search or study selection process.

### Data Extraction and Quality Assessment

The following information was extracted from the included studies: study name; subject characteristics, including sample size, percentage of women and mean age at baseline; wine consumption; grams of ethanol consumed per day; type of wine consumed; test used to measure cognitive function and cognitive domains measured.

To evaluate the risk of bias of cohort studies, we used the Quality Assessment Tool for Observational Cohort and Cross-Sectional Studies from the United States National Institute of Health National Heart, Lung, and Blood Institute (36). This tool assesses 14 items for longitudinal studies in the following domains: (1) research question, (2) study population, (3) participation rate, (4) recruitment, (5) sample size, (6) timeframe for associations, (7) exposure levels, (8) exposure measures and (9) assessment, (10) repeated exposure assessment, (11) outcome measures, (12) blinding of exposure assessors, (13) loss to follow-up, and (14) statistical analyses.

For case-control studies, the Quality Assessment of Case-Control Studies (37) from the United States National Institute of Health National Heart, Lung, and Blood Institute was used. This tool includes 12 domains, namely: (1) research question, (2) study population, (3) target population, (4) sample size, (5) recruitment, (6) inclusion and exclusion criteria, (7) case and control definitions, (8) random selection of study participants, (9) concurrent controls, (10) exposure measures and assessment, (11) blinding of exposure assessors, and (12) statistical analyses.

Finally, the overall risk of bias of each study was scored as “good” if most criteria were met; “fair” if some criteria were met; or “poor” if few criteria were met.

The search strategy, study selection, eligibility, data extraction, and quality assessment, were conducted by two independent reviewers (M.L.-L.-T and C.A.-B.). When the agreement was not reached, a third reviewer (I.C.-R.) was consulted.

### Statistical Analysis and Data Synthesis

A meta-analysis was performed to determine the association between wine consumption and cognitive decline. The studies were classified according to wine consumption in the three subgroups, namely: (i) “within WHO recommendations” when studies reported wine consumption of <20 g in women and 30 g in men; (ii) “above the WHO recommendations” when studies reported higher wine consumption; and (iii) “unclassified” when studies did not report participants' wine consumption.

Some methodological considerations should be noted. When two studies reported data from the same population, we include in the meta-analysis the study with the largest sample size. The RR and odds ratio (OR) for the association between wine consumption and cognitive decline were jointly included in the meta-analysis (38). When studies reported the hazard ratio (HR), it was converted to RR using the following formula: RR=(1 – *e*HRln(1 – *r*))/*r* (38). In addition, the type and consumption of wine were collected as reported by the original studies and converted into grams using the equivalences of the SBU.

The DerSimonian and Lair and Hartung-Knapp-Sidik-Jonkman random-effects methods were used to compute the pooled estimate of the RR and their respective 95% Cis (39, 40). Following the Cochrane Handbook recommendations, the *I*^2^ statistic was used to examine the inconsistency, which ranges between 0 and 100% (41). According to the *I*^2^ values, inconsistency was considered not important (0–30%), moderate (≥30–50%), substantial (≥50–75%), or considerable (≥75–100%). The corresponding *p*-values were also considered. In addition, heterogeneity was evaluated using the τ^2^ statistic, which was interpreted as low when τ^2^ was lower than 0.04, moderate when τ^2^ was from ≥0.04 to 0.14, and substantial when τ^2^ was from ≥0.14 to 0.40 (42).

To assess the robustness of the summary estimates, sensitivity analyses were conducted by removing each study one at a time from the pooled estimations. Meta-regression analyses were performed to address whether mean age, percentage of women, and time of follow-up, as continuous variables, could modify the effect of the association between wine consumption and cognitive decline. Finally, publication bias was assessed through Egger's regression asymmetry test, where a *p*-value of <0.10 was used to determine if there was significant publication bias (43). Analyses were performed using Stata 15.0 (Stata, College Station, TX, USA).

## Results

### Study Selection

**Figure 1:**
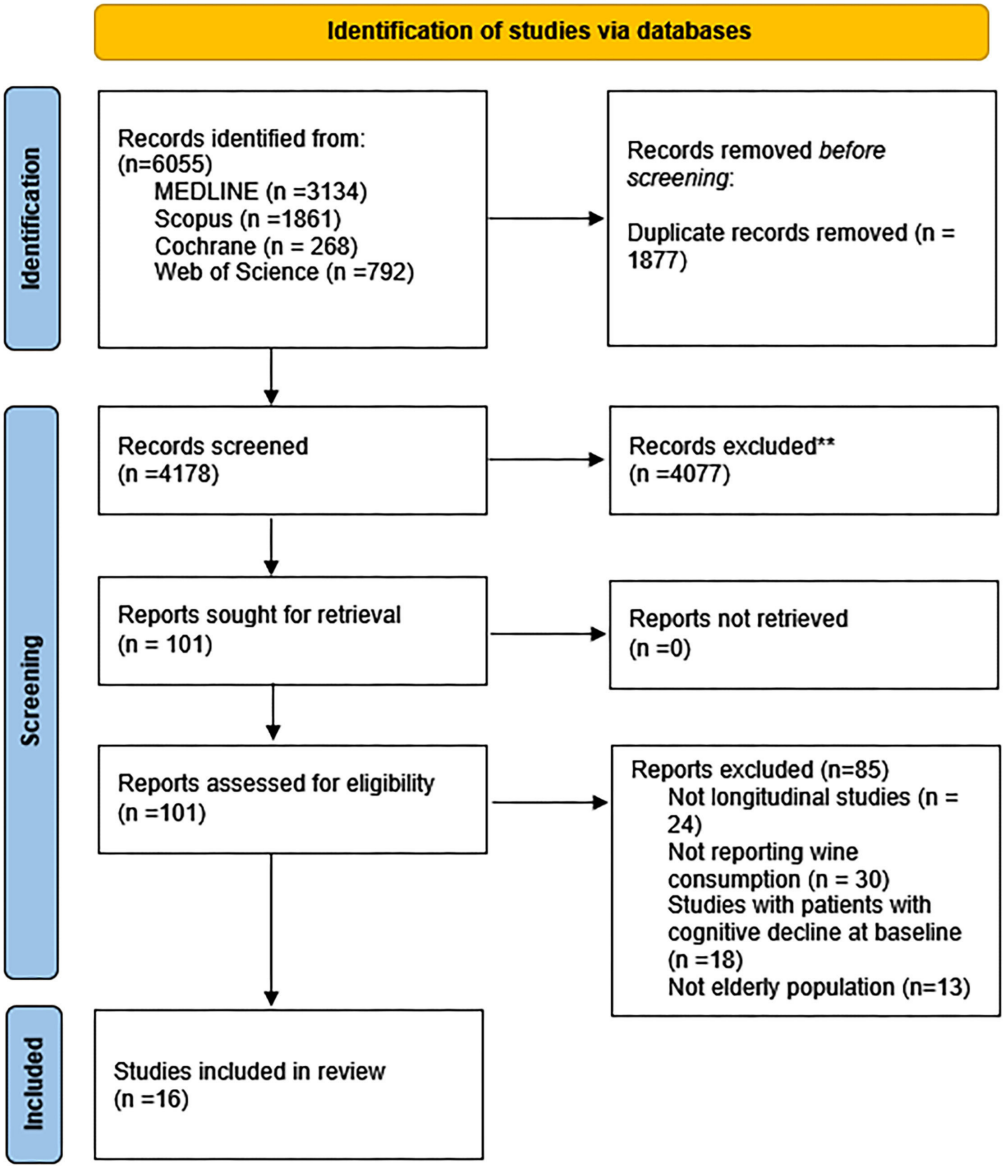
PRISMA 2020 flow diagram for new systematic reviews which included searches of databases.

The search retrieved 6,055 articles. From them, 101 studies were selected by reviewing the title and abstract, and 16 of which were included in this systematic review (25, 26, 44–58). Only 12 of these studies were included in the meta-analysis (Figure 1) (25, 26, 44–48, 50, 52, 53, 55, 58).

### Study and Intervention Characteristics

The included longitudinal studies (25, 26, 44–58) were published between 1997 and 2019. They were conducted in ten different countries, including China (1 study), Canada (1 study), Germany (1 study), Sweden (2 studies), the United States (4 studies), the Netherlands (2 studies), France (2 studies), the United Kingdom (1 study), Italy (1 study), and Denmark (1 study).

The sample size of the included studies ranged from 360 to 10,308, with a total of 46,472 participants (60% women) aged 70 years old. Their follow-up periods ranged from 2 to 43 years. All the studies included the general population without dementia at baseline (25, 26, 44–58). One of the studies was conducted on twins born in Sweden between 1907 and 1925 (46), and another included only women (53). Only one study (54) did not report wine consumption and only three reported the type of wine consumed, distinguishing among white, red, and fortificated (Table 1) (41, 47, 51).

**Table 1:** Main characteristics of the included studies.

**References**	**Exposure**				**Outcomes**				
	**N, (women %); wine, (women %)**	**Ages (DE)**	**Follow-up (years)**	**Wine consumption**	**Equivalent gr/day etanol in wine**	**Type of wine**	**Cognition test**	**Cognition domain**
Deng et al. (44) China	2,632 (56.38); 123	Non-drinker: 69.2 (8.8) Light-to-moderate drinker: 66.3 (6.3) Excessive drinker: 65.2 (7.7)	2	- Non-drinker (<1U/w) - Light-to-moderate drinker (1–21 U/w ♂, or 1–14 U/w ♀) - Excessive drinker (>21 U/w ♂, or >14 U/w ♀))	- Non-drinker: <1,43 gr - Light-to-moderate drinker: 1,43–30 gr ♂, 1,43–20 gr ♀) - Excessive drinker (>30 gr ♂, or >20 gr ♀)	Wine	MMSE Suspected dementia: - Examination by a neurologist - neuropsychological testing	- Dementia.
Fischer et al. (45) Germany	2,622 (65.3)	81.2 (3.4)	10	Red wine (%): - Never: 52.2 - <1 time/week: 20.4 - 1 time/week: 9.2 - Several times/week: 10.7 - Every day: 7.5 White wine (%): - Not at all :64.4 - <1 time/week:20.6 -1 time/week: 6.1 - Several times/week: 7.0 - Every day: 1.9	NR	Red wine White wine	SIDAM Global Deterioration Scale Blessed Dementia Rating scale CERAD: - Word List Immediate Recall - Word List Delayed Recall - Word List Recognition subtest	- AD. - Dementia - Dementias. - Memory.
Handing et al. (46) Sweden	12,326 (55.5); 5,463	54.2 (5.9)	43	n (mean) - None (0): 31 (0.0) - Light (>0 to ≤ 5): 5,102 (0.5) - Moderate (>5 to ≤ 12): 255 (6.3) - Heavy (>12 to ≤ 24): 56 (18.9) - Very Heavy (>24): 19 (33.6)	- None: 0 gr - Light: 0–5 gr - Moderate: 5–12 gr - Heavy: 12 gr-24 gr - Very Heavy >24 gr	Wine	The National Patient Register and/or Cause of Death Register were used for diagnoses of dementia.	- Dementia.
Heymann et al. (47) EEUU	360 (58)	74.87 (8.897)	19.28	- Abstainers - Mild-moderate drinkers (1–7 D/w). - Heavy drinkers (8 or more D/w).	- Abstainers: 0 - Mild-moderate drinkers:11–77 gr - Heavy drinkers: >88 gr	Wine	MMSE.	- AD.
Leibovici et al. (48) France	833	>60	3	Below 75 years: - Low education: 26% - High education: 17% >75: - Low education: 47% - High education: 27%	NR	Wine	- Examen Cognitif par Ordinateur	- Working memory. - Language skills. - Visuospatial performance. - Focused and divided attention.
Lemeshow et al. (49) EEUU	3,777	≥65	3	Incident dementia. N (%) - None: 48 (4.9) - ≤ 1/4 liter/day: 47 (5.1) - >1/4 liter/day: 4 (1.1)	- ≤ 1/4 litter/day: 0–24 gr - >1/4 litter/day: >25 gr	Wine	Battery of psychometric test: - The Benton Visual Retention Test. - The Zazzo's Cancellation Test. - The Isaacs Stet Test for verbal fluency. - The Wechsler paired associate's test. - The Wechsler digit-symbol test. - MMSE. - DSM-III-R criteria.	- Cognitive functioning. - Global cognitive status. - Dementia. - AD.
Lindsay et al. (50) Canadá	Cases: 194 (67.5) Controls: 3894 (57.5)	Cases: 81 Controls: 72.9	5	- Cases exposed/total: 15/186 - Controls exposed/total: 668/3,789	NR	Wine	- 3MSE. - Neuropsychological tests	- AD
Low et al. (51) France	9,294 (66)	75.8 (4.35)	12	Wine (glasses/week) Cases: 8.4 (9.5) Controls: 9.3 (11.5)	NR	Wine Red wine	Mini-Mental State Examination. Benton Visual Retention Test. Isaac's Set Test. Trail-Making Test part A. Trail-Making Test part B.	Global cognition.
Luchsinger et al. (52) EEUU	980 (67); 162	73.3 (5.8) Age according to the frequency of consumption: None: 75.37 (5.78) Light to moderate: 75.11 (5.62) Heavy: 82.41 (0)	4	None:85,91% Light to moderate: 14,08% Heavy: 0,10%	None: 0 gr Light to moderate: 11–33 gr Heavy: >33 gr	Wine	Alzheimer's disease: - National Institute of Neurological and Cognitive Disorders and Stroke-Alzheimer's Disease and Related Disorders Association criteria. Dementia: - Diagnostic and Statistical Manual of Mental Disorders, Fourth Edition criteria and required evidence of cognitive deficit on the neuropsychological test battery and evidence of impairment in social or occupational function; persons with a global summary score on the Clinical Dementia Rating (CDR).	- AD. - Dementia.
Mehlig et al. (53) Sweden	Four health examinations - 1968–1969: 1,458 - 1974–1975 (alive): 1,298 (1,426) - 1980–1981 (alive): 1,146 (1,366) - 1992–1993 (alive): 800 (1,118)	38–60 years	34	Four health examinations: - 1968–1969: 51% - 1974–1975: 59% - 1980–1981: 63% - 1992–1993: 64%	NR	Wine	Batteries of neuropsychiatric tests.	- Dementia.
Mukamal et al. (26) EEUU	746 (58,45)	77,65 (5.35)	6	- None - <1 - 1–6 - >o=7	- None: 0gr - <1: <2,35 gr - 1–6: 2,36gr-14,14 gr - >7: >14,14	Wine	- 3MSE - The CHS cognition study - IQCODE.	- Dementia.
Nooyens et al. (54) The Netherlands	2,613 (50.71);	55.78 (7)	7	NR	NR	White wine Red wine Fortified wine	The neuropsychological test battery included four tests: - The 15 Words Verbal Learning Test. - The Stroop Color-Word Test. - The Word Fluency Test. - The Letter Digit Substitution Test.	- Memory. - Speed and cognitive flexibility. - Semantic memory. - Speed.
Orgogozo et al. (55) France	3,777	73,6	3	- Non-drinkers: 0– <1 per week. - Mild drinkers: At least 2 drinks per week but not more than 250 ml per day. - Moderate drinkers: 250–500 ml per day. - Heavy drinkers: >500 ml per day.	- Non-drinkers: 0 mg. - Mild drinkers: no more than 25 gr. - Moderate drinkers: 25–50 gr. - Heavy drinkers: >50 gr.	Wine	Battery of psychometric test: - The Benton Visual Retention test. - The Zazzo's Cancellation Test. - The Isaacs Stet Test for verbal fluency. - The Wechsler paired associates test. - The Wechsler digit-symbol test. - MMSE. - DSM-III-R criteria.	-Cognitive functioning. - Global cognitive status. - Dementia. - AD.
Ruitenberg et al. (56) Netherlands	5,395 (59); 1,994 (42)	67,38 (7,48)	6	Median (IQR) drinks/day: -Total (*n* = 1,994): 0·14 (0·05–0·47) Men (*n* = 655): 0·24 (0·06–0·59) Women (*n* = 1,339): 0·12 (0·04–0·44)	NR	Wine	- MMSE	- Dementia.
Sabia et al. (57) UK	10,308 (33)	35-55	31	Units/week (%) - Abstinence: 0 (0) - 1–14 units/week: 2.8 (2.5) - >14 units/week7.9 (7.1)	NR	Wine	The national hospital episode statistics. The Mental Health Services Data Set. The mortality registers. Using ICD-10 codes F00-F03, F05.1, G30, and G31.	- Dementia.
Solfrizzi et al. (58) Italy	2,963; 1,131 (36.25)	71,69 (4.965) Alcohol consumption None: 71.09 (4.94) <1 drink/day: 72.12 (5.06) 1 or 2 drinks/day: 71.90 (5.06) >2 drinks/day: 71.67 (4.80)	3,5	Median (IQR) drinks/day: - Total: 1.69 (0.85–1.69) ♂: 1.69 (0.85–3.38) ♀: 0.85 (0.85–0.85)	- None - <1: <11,5 gr - 1–2: 12,5gr-24gr - >2: >25 gr	Wine	MMSE.	- Dementia. - Cognitive impairment.
Truelsen et al. (25) Denmark	Total: 1,709 (62.14) Cases: 83 (54.21) Controls: 1,626 (62.54)	Total: 75.8 (6) Cases: 73.3 (5.6) ♀: 78.9 (5.8) ♂: 77.6 (7.1) Controls: 78.3 (6.4) ♀: 73.3 (5.5) ♂: 73.2 (5.6)	3	% ♂; % ♀ - Never/hardly ever: 60.5; 62.2 - Monthly: 26.3; 26.7 - Weekly: 7.9; 8.9 - Daily: 5.3; 2.2	NR	Wine	MMSE.	- Dementia.

*U/w, unit per week; D/w, drinks of alcohol per week; NR, not reported; MMSE, Mini-Mental State Examination; AD, Alzheimer's disease; DSM, Diagnostic and Statistical Manual of Mental Disorders; IQCODE, Informant Questionnaire on the Cognitive Decline of the Elderly; CHS, Health Study-Cognition; 3MSE, Modified Mini-Mental State; CERAD, the Consortium to Establish a Registry for Alzheimer's Disease neuropsychological battery*.

Finally, the most used tests to assess the risk of cognitive decline or dementia were the Mini-Mental State Examination (MMSE), the SIDAM test, the Global Deterioration Scale and the Blessed Dementia Rating Scale, and the Consortium to Establish a Registry for Alzheimer's Disease (CERAD) neuropsychological battery. Other tests used were the Battery of psychometric test, the Diagnostic and Statistical Manual of Mental Disorders (DSM-III-R Criteria), the Modified Mini-Mental State (3MSE), the Health Study**-**Cognition (CHS), and the Informant Questionnaire on the Cognitive Decline of the Elderly (IQCODE) (Table 1).

### Risk of Bias

For cohort studies, the overall risk of bias was low in all studies (100%). All the studies included information on the domains related to exposure levels and the time frame to observe an effect. Only two studies (45, 48) provided information on the blinding of the assessors (13.32%). In addition, the participation rate was not reported in 42.86% of studies, and the follow-up rate was not achieved in 35.71%. Finally, only one study (48) did not provide information on the exposure measures (6.66%) and only 28.6% of studies assessed the exposure on more than one occasion (Supplementary Table 2).

For case-control studies, the overall risk of bias was low in all studies (100%) (25, 50). The studies provided information on all the domains, except one study (50) that did not report information about blinding of exposure assessors (50%) (Supplementary Table 3).

### Meta-Analysis

**Figure 2:**
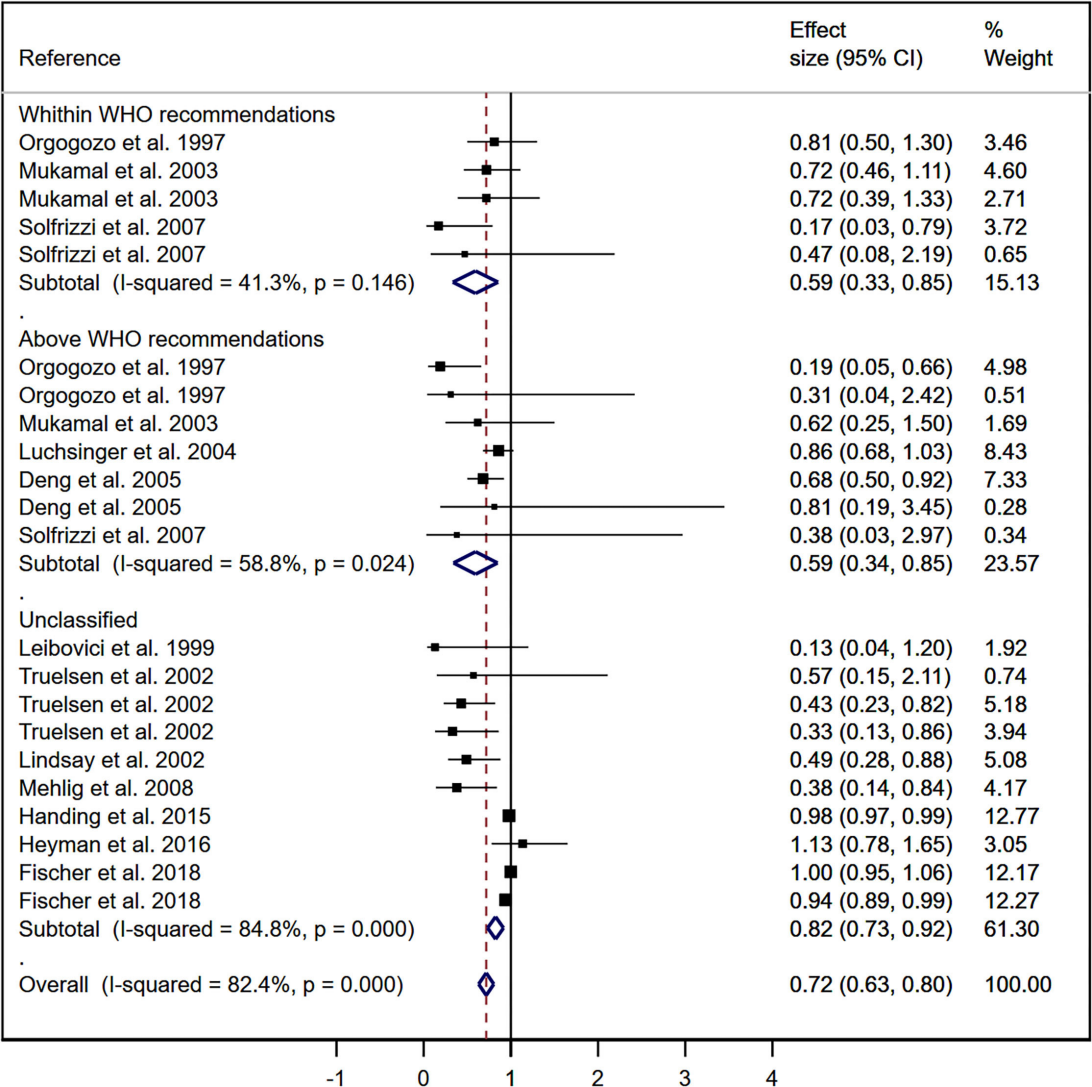
Meta-analysis for the association of wine consumption and cognitive impairment.

Using the DerSimonian and Lair random effect models, the pooled RR for the association of wine consumption with cognitive decline was 0.72 (95% CI 0.63–0.80; *I*^2^ = 82.4%; τ^2^: 0.0154). For the subgroup analysis, the pooled RR in those cohorts within the WHO recommendations was 0.59 (95% CI: 0.33–0.85; *I*^2^ = 41.3%; τ^2^: 0.0348), the pooled RR for those cohorts above the WHO recommendations was 0.59 (95% CI: 0.34–0.85; I^2^ = 58.8%; τ^2^: 0.042), and the pooled RR for unclassified cohorts was 0.82 (95% CI: 0.73–0.92; *I*^2^ = 84.8%; τ^2^: 0.0088) (Figure 2).

Using the Hartung-Knapp-Sidik-Jonkman random effect models, the pooled RR for the association of wine consumption with cognitive decline was 0.65 (95% CI 0.52–0.79; *I*^2^ = 94,531%; τ^2^: 0.057). For the subgroup analysis, the pooled RR for those cohorts within the WHO recommendations was 0.59 (95% CI: 0.32–0.87; *I*^2^ = 45.87%; τ^2^: 0.042), the pooled RR for those cohorts above the WHO recommendations was 0.59 (95% CI: 0.32–0.86; *I*^2^ = 63.9%; τ^2^: 0.060), and the pooled RR for unclassified cohorts was 0.68 (95% CI: 0.47–0.90; *I*^2^ = 98.27%; τ^2^: 0.091).

### Sensitivity and Meta-Regression Analysis

The pooled RR estimates were not modified after removing one study at a time from the analyses (Supplementary Table 4). In addition, random-effects meta-regression models for the association between wine consumption and cognitive decline revealed that age, percentage of women, and duration of follow-up were not related to heterogeneity across studies (Supplementary Table 5).

### Publication Bias

Publication bias was observed by Egger's test (43) for the overall effect of wine consumption on cognitive decline (*p* < 0.01). For subgroup analyses, publication bias was found for the unclassified cohorts (*p* < 0.01).

## Discussion

This systematic review and meta-analysis provide an overview of the evidence on the association of wine consumption with cognitive decline. Our data support the association between wine consumption and a lower risk of cognitive decline, with this evidence being stronger for those populations within WHO recommendations (30 g for men and 20 g for women, i.e., 3 SBUs and 2 SBUs, respectively) (14). Finally, this association did not seem to be modified by mean age, percentage of women, or follow-up time. Our results are in accordance with previous research (9, 22, 59) reporting the effect of alcohol consumption, including wine in sub-analyses, and add to the evidence that a protective effect could be exerted by low-to-moderate wine consumption.

Many lifestyle habits have been proposed to prevent or delay cognitive decline, including physical exercise, socialization, cognitive training, maintenance of good sleep hygiene, and adoption of the Mediterranean diet, among others. It has been reported that the Mediterranean diet could help to control cardiovascular parameters and oxidative stress. The Mediterranean diet contains a wide variety of foods rich in antioxidants, including a low-to-moderate wine consumption (60–62). This moderate alcohol consumption has been associated with better cognitive performance (63) and may contribute to maintaining cognition levels, although these results should be interpreted with caution since some types of alcohol-associated dementia have been identified (64, 65).

It is usually accepted that the limits for healthy alcohol consumption are 30 g for men and 20 g for women (14). Within these limits, most literature supports the possible protective effect of wine consumption against cognitive decline, even after controlling for covariates, such as age and percentage of women. However, some studies found no difference in the association of wine consumption and cognitive decline between women and men (25, 26, 44–58), Although, our data support no differences by sex, others have reported a higher association for women (45, 66) due to the differences in wine consumption between groups (45) and women's preference for white wine (33).

Wine consumption has been associated with a reduced risk of cognitive decline due to some of the components present in wine that may have an antioxidant function or that could inhibit the lethal events of oxidative stress produced by nitric oxide (24, 32). Grape skins are made up of different types of polyphenols, namely, quercetin, myricetin, catechin, and epicatechin (flavonols), gallic acid, and polymeric anthocyanins (24). The concentration of these components, which varies among types of wine, is responsible for the promising antioxidant potential of wine, with some wines being more protective than others. Flavonoids are components present primarily in red wine that might explain the reduction in the incidence of Alzheimer's disease and cerebrovascular disease (32, 66, 67). Resveratrol, a plant compound found in red wine grapes, might appear to have a neuroprotective effect by protecting neuronal cells from β-amyloid, a neurotoxin involved in the creation of senile plaques detected in neurodegenerative diseases, such as Alzheimer's disease. These senile plaques contribute to cell death (68), although removal of these plaques does not lead to improved cognition (69), resveratrol has anti-neuroinflammatory properties that protect against cognitive decline (57). This substance also has cancer-inhibiting effects (70) and reduces the incidence of coronary heart disease (71).

Moreover, there are also phenolic compounds in wine that contribute to the sensory properties of wine and protect it from oxidation (72, 73). These compounds also have antioxidant effects, reducing the risk of degenerative diseases such as osteoporosis, diabetes, and cancer (74). Various *in vitro* and animal studies have shown that polyphenols such as resveratrol could exert a number of health benefits, including anti-inflammatory and anti-atherogenic effects (27), and suggest a possible use of these polyphenols as therapeutic agents for ischemic and neurodegenerative events (75). In humans, a randomized clinical trial in women using a resveratrol supplement, concluded that a low-dose resveratrol could be a preventive strategy to counteract aging factors such as cognitive decline (76). Furthermore, it has been reported that resveratrol consumption needed for improvements in cognitive functions is far from the daily intake associated with food and wine consumption (77), A very high wine consumption, that would be detrimental to health, would be necessary to achieve the cognitive healthy resveratrol intake (78). In the case of observational studies with polyphenol supplementation through diet, including polyphenol-rich foods such as vegetables, fruit, or wine consumption (79), a low-dose wine consumption could produce benefits for some pathologies (53). In addition, evidence suggests that carrying the apolipoprotein E (*APOE*) epsilon 4 allele increases the likelihood of developing Alzheimer's disease (80). The risk of Alzheimer's disease in *APOE* ε4 carriers increased with white wine consumption vs. red wine consumption (41). Furthermore, the possible benefits of moderate wine consumption are increased in *APOE* ε4 noncarriers (6, 81). These possible benefits of wine components could explain the famous French paradox, where it was observed that mortality from CVD was much lower in France than in other industrialized countries (81). Further studies should consider additional confounding variables, namely, age, sex, race, body mass index, smoking, marital status, education, hormone treatment, and some pathologies such as diabetes (23).

Our systematic review and meta-analysis have some limitations that should be mentioned. First, studies included a wide variety of scales for measuring cognitive decline, which can lead to bias. Second, each study measured wine consumption differently, and some of the included studies that did not quantify wine consumption. Third, there is no global consensus on maximum recommended intake or safe drinking limits, so the use of alcohol consumption limits, although recommended by the WHO, could represent a limit in addressing this issue. Fourth, most studies did not provide information on assessor blinding or whether exposure was assessed more than once over time. Fifth, we found evidence of publication bias using the Egger's test. Sixth, we only included studies in English and Spanish, in addition, gray literature was not included. Finally, due to the lack of data, we were unable to analyze the association between wine consumption and cognitive decline by type of wine.

In summary, this systematic review and meta-analysis identified a possible protective effect of wine consumption on the development of cognitive decline. This effect appears to be independent of age, the percentage of women, and follow-up time. These results do not suggest that the population should increase their wine consumption, as this could be harmful, especially in older persons who, with the loss of lean mass, polymedication and other factors, could suffer serious harm. However, considering our results, low wine consumption could be promoted within other lifestyles, including the Mediterranean diet, as an effective habit to prevent or delay cognitive deterioration in the healthy population. These results also support the international guidelines that suggest low-to-low-moderate alcohol consumption as the most acceptable level of consumption, both in the long and short term and for people who do not suffer any pathology such as liver cirrhosis or are not polymedicated or pregnant (82–84). Finally, it would be important for future research to differentiate between white and red wine consumption, which could allow to determine the association between wine consumption and cognitive decline by type of wine.

## Data Availability Statement

The original contributions presented in the study are included in the article/Supplementary Material, further inquiries can be directed to the corresponding author.

## Author Contributions

ML-L-T and CÁ-B: conceptualization, investigation, and writing—original draft preparation. ML-L-T, CÁ-B, and IC-R: methodology. IC-R and CÁ-B: software. AS-L and CP-M: validation and visualization. ML-L-T and IC-R: formal analysis. ML-L-T, AS-L, and CP-M: resources. CÁ-B and VM-V: data curation. VM-V: writing—review and editing. CÁ-B: supervision. All authors revised and approved the final version of the articles.

## Funding

This work was funded by FEDER funds.

## Conflict of Interest

The authors declare that the research was conducted in the absence of any commercial or financial relationships that could be construed as a potential conflict of interest.

## Publisher's Note

All claims expressed in this article are solely those of the authors and do not necessarily represent those of their affiliated organizations, or those of the publisher, the editors and the reviewers. Any product that may be evaluated in this article, or claim that may be made by its manufacturer, is not guaranteed or endorsed by the publisher.
