# Supplementary material for: Association Between Wine Consumption and Cognitive Decline in Older People: A Systematic Review and Meta-Analysis of Longitudinal Studies
Source: Front Nutr. 2022 May 12;9:863059. doi: 10.3389/fnut.2022.863059 (PMC9133879; doi:10.3389/fnut.2022.863059)
Supplement: Supplementary file 1 [file Table_1.docx]

**Table S1.** Complete search strategy for MEDLINE.

| **Population** | **Intervention** | **Outcome** |
| --- | --- | --- |
| Older  OR  Elderly  OR  Elderly people  OR  Older people | Alcohol  OR  Wine  OR  Alcohol consumption  OR  Wine consumption | Dementia  OR  Mental deterioration  OR  Alzheimer disease  OR  Vascular dementia  OR  Predementia syndromes  OR  Mild cognitive impairment |

| **References** | **1** | **2** | **3** | **4** | **5** | **6** | **7** | **8** | **9** | **10** | **11** | **12** | **13** | **14** | **Quality** |
| --- | --- | --- | --- | --- | --- | --- | --- | --- | --- | --- | --- | --- | --- | --- | --- |
| Deng et al. | Y | Y | Y | Y | Y | Y | Y | Y | Y | N | Y | NR | Y | Y | Good |
| Fischer et al. | Y | Y | Y | Y | Y | Y | Y | Y | Y | N | Y | N | Y | Y | Good |
| Handing et al. | Y | Y | NR | Y | Y | Y | Y | Y | Y | N | Y | NR | NR | Y | Good |
| Heymann et al. | Y | Y | NR | Y | Y | Y | Y | Y | Y | N | Y | NR | Y | Y | Good |
| Leibovici et al. | Y | Y | Y | Y | Y | Y | Y | N | Y | N | Y | Y | Y | Y | Good |
| Lemeshow et al. | Y | Y | Y | Y | Y | Y | Y | Y | Y | Y | Y | NR | NR | Y | Good |
| Luchsinger et al. | Y | Y | Y | Y | Y | Y | Y | Y | Y | N | Y | NR | N | Y | Good |
| Mehlig et al. | Y | Y | Y | Y | Y | Y | Y | Y | Y | N | Y | NR | NR | Y | Good |
| Mukamal et al. | Y | Y | NR | Y | Y | Y | Y | Y | Y | N | Y | NR | Y | Y | Good |
| Nooyens et al. | Y | Y | Y | Y | Y | Y | Y | Y | Y | N | Y | NR | Y | Y | Good |
| Orgogozo et al. | Y | Y | Y | Y | Y | Y | Y | Y | Y | Y | Y | NR | Y | Y | Good |
| Ruitenberg et al. | Y | Y | NR | Y | Y | Y | Y | Y | Y | Y | Y | NR | Y | Y | Good |
| Sabia et al. | Y | Y | NR | Y | Y | Y | Y | Y | Y | Y | Y | NR | NR | Y | Good |
| Solfrizzi et al. | Y | Y | NR | Y | Y | Y | Y | Y | Y | N | Y | NR | NR | Y | Good |

**Table S2.** Risk of bias assessment table using Quality Assessment Tool for Observational Cohort and Cross-Sectional Studies.

1. Was the research question or objective in this paper clearly stated?; 2. Was the study population clearly specified and defined?; 3. Was the participation rate of eligible persons at least 50%?; 4. Were all the subjects selected or recruited from the same or similar populations (including the same time period)? Were inclusion and exclusion criteria for being in the study prespecified and applied uniformly to all participants?; 5. Was a sample size justification, power description, or variance and effect estimates provided?; 6. For the analyses in this paper, were the exposure(s) of interest measured prior to the outcome(s) being measured?; 7. Was the timeframe sufficient so that one could reasonably expect to see an association between exposure and outcome if it existed?; 8. For exposures that can vary in amount or level, did the study examine different levels of the exposure as related to the outcome (e.g., categories of exposure, or exposure measured as continuous variable)?; 9. Were the exposure measures (independent variables) clearly defined, valid, reliable, and implemented consistently across all study participants?; 10. Was the exposure(s) assessed more than once over time?; 11. Were the outcome measures (dependent variables) clearly defined, valid, reliable, and implemented consistently across all study participants?; 12. Were the outcome assessors blinded to the exposure status of participants?; 13. Was loss to follow-up after baseline 20% or less?; 14. Were key potential confounding variables measured and adjusted statistically for their impact on the relationship between exposure(s) and outcome(s)?; N: no; NR: not reported; Y: yes.

**Table S3.** Risk of bias assessment table using Quality Assessment Tool for Case-Control Studies

| **References** |  | | **Items** | | | | | | | | | | | | **Quality** | |
| --- | --- | --- | --- | --- | --- | --- | --- | --- | --- | --- | --- | --- | --- | --- | --- | --- |
|  | **1** | **2** | | **3** | **4** | **5** | **6** | **7** | **8** | **9** | **10** | **11** | **12** |  | |  |
| Truelsen et al. | Y | Y | | Y | Y | Y | Y | Y | Y | Y | Y | Y | Y | Good | |  |
| Lindsay et al. | Y | Y | | Y | Y | Y | Y | Y | Y | Y | Y | NR | Y | Good | |  |

1.Was the research question or objective in this paper clearly stated and appropriate?; 2. Was the study population clearly specified and defined?; 3. Did the authors include a sample size justification?; 4. Were controls selected or recruited from the same or similar population that gave rise to the cases (including the same timeframe)?; 5. Were the definitions, inclusion and exclusion criteria, algorithms or processes used to identify or select cases and controls valid, reliable, and implemented consistently across all study participants?; 6. Were the cases clearly defined and differentiated from controls?; 7. If less than 100 percent of eligible cases and/or controls were selected for the study, were the cases and/or controls randomly selected from those eligible?; 8. Was there use of concurrent controls?; 9. If matching was used, did the investigators account for matching during study analysis?; 10. Were the measures of exposure/risk clearly defined, valid, reliable, and implemented consistently (including the same time period) across all study participants?; 11. Were the assessors of exposure/risk blinded to the case or control status of participants?; 12. Were key potential confounding variables measured and adjusted statistically in the analyses?; N: no; NR: not reported; Y: yes.

**Table S4.** Sensitivity analysis.

| **Within WHO recommendations** | | | |
| --- | --- | --- | --- |
| **Reference** | **RR** | **LL** | **UL** |
| Orgogozo et al. 1997 | 0,528 | 0,217 | 0,840 |
| Mukamal et al. 2003 | 0,546 | 0,196 | 0,896 |
| Mukamal et al. 2003 | 0,561 | 0,232 | 0,896 |
| Solfrizzi et al. 2007 | 0,736 | 0,518 | 0,953 |
| Solfrizzi et al. 2007 | 0,601 | 0,309 | 0,893 |

| **Above WHO recommendations** | | | |
| --- | --- | --- | --- |
| **Reference** | **RR** | **LL** | **UL** |
| Orgogozo et al. 1997 | 0,770 | 0,641 | 0,900 |
| Orgogozo et al. 1997 | 0,604 | 0,340 | 0,871 |
| Mukamal et al. 2003 | 0,586 | 0,299 | 0,872 |
| Luchsinger et al. 2004 | 0,488 | 0,234 | 0,743 |
| Deng et al. 2005 | 0,544 | 0,154 | 0,933 |
| Deng et al. 2005 | 0,586 | 0,320 | 0,852 |
| Solfrizzi et al. 2007 | 0,598 | 0,332 | 0,864 |

| **Unclassified** | | | |
| --- | --- | --- | --- |
| **Reference** | **RR** | **LL** | **UL** |
| Leibovici et al. 1999 | 0,850 | 0,763 | 0,937 |
| Truelsen et al. 2002 | 0,826 | 0,734 | 0,917 |
| Truelsen et al. 2002 | 0,870 | 0,785 | 0,956 |
| Truelsen et al. 2002 | 0,865 | 0,780 | 0,950 |
| Lindsay et al. 2002 | 0,860 | 0,773 | 0,948 |
| Mehlig et al. 2008 | 0,863 | 0,777 | 0,949 |
| Handing et al. 2015 | 0,690 | 0,539 | 0,842 |
| Heyman et al. 2016 | 0,811 | 0,717 | 0,904 |
| Fischer et al. 2018 | 0,729 | 0,605 | 0,853 |
| Fischer et al. 2018 | 0,733 | 0,604 | 0,863 |

**Table S5.** Meta-regression analysis.

|  | | Coefficient | 95%ICs | | I^2^ | | P value |
| --- | --- | --- | --- | --- | --- | --- | --- |
| Whithin WHO recommendations | | | | | | | |
| Mean age | -0.108 | | | -0.256, 0.039 | 0.00% | 0.102 | |
| % females | -0.011 | | | -0.061, 0.040 | 43.64% | 0.554 | |
| Follow-up | 0.066 | | | -0.288, 0.420 | 48.60% | 0.595 | |
| Above WHO recommendations | | | | | | | |
| Mean age | | -0.018 | -0.113, 0.076 | | 65.16% | | 0.638 |
| % females | | 0.012 | -0.003, 0.028 | | 9.14% | | 0.094 |
| Follow-up | | 0.034 | -0.276, 0.343 | | 61.94% | | 0.790 |
| Unclasiffied | | | | | | | |
| Mean age | | 0.009 | -0.018, 0.035 | | 85.98% | | 0.463 |
| % females | | -0.010 | -0.031, 0.096 | | 83.85% | | 0.255 |
| Follow-up | | 0.009 | -0.009, 0.026 | | 85.16% | | 0.290 |
